# JKBNS's Scopus indexing: a leap toward the advancement of biological nursing science

**DOI:** 10.7586/jkbns.25.010

**Published:** 2025-02-25

**Authors:** Myoung-Ae Choe

**Affiliations:** Professor emeritus, Seoul National University College of Nursing, Seoul, Korea

As the founding president of the Korean Society of Biological Nursing Science (KSBNS), I was profoundly gratified to learn that the *Journal of Korean Biological Nursing Science* (JKBNS) has been indexed in Scopus. To mark this milestone, I would like to reflect on the 26-year journey that brought us here.

The KSBNS was formally established in 1998. However, its origins trace back to a collaborative research group formed by professors specializing in basic medical sciences—including anatomy, physiology, and pharmacology—and those teaching in related fields. In December 1997, this group convened its inaugural meeting, initiating monthly discussions to develop a biological nursing curriculum aligned with nursing practice and to organize educational content.

The professors participating in the research group were driven by passion and a shared mission: to establish biological nursing science as a distinct discipline and to create a curriculum tailored to nursing’s practical demands. Their collective resolve stemmed from a belief that biological nursing science must form the bedrock of nursing education and research.

A year after the research group’s formation, the KSBNS was officially launched in December 1998. This institutionalization marked a pivotal step in advancing biological nursing science, providing structure for the discipline’s growth and fostering collaboration among scholars.

To solidify the field’s academic standing, the KSBNS published its inaugural journal issue in December 1999. Initially released biannually (2000–2009), the journal transitioned to triannual publication in 2010 and quarterly in 2013. These efforts provided a dedicated platform for disseminating research, promoting dialogue, and building a cohesive knowledge base for the discipline.

A study by Lee et al. [1] analyzed 179 papers published in JKBNS from its first issue in 1999 to 2010, revealing annual increases in output, diversification of topics and methodologies, and a higher proportion of experimental studies compared to other nursing journals. Crucially, the majority of these studies provided evidence for clinical nursing practice, contributing to evidence-based nursing.

JKBNS achieved indexing in the Korea Citation Index in 2010 and in Cumulative Index to Nursing and Allied Health Literature (CINAHL) in 2012. After 12 years in CINAHL, its 2024 inclusion in Scopus—a multidisciplinary database with rigorous evaluation standards—has elevated the journal’s global visibility. This milestone enables worldwide access to its content and invites international submissions, cementing JKBNS as a journal of international reach.

This achievement stems from sustained efforts since 2012: quarterly publication, the 2014 launch of an English-language website and submission system, and rising English manuscript submissions. Equally vital has been the dedication of reviewers, editors, editor-in-chief, and society leaders, as well as the support and cooperation of past presidents and executives. I extend my heartfelt congratulations and appreciation to all contributors for their indispensable role in this success.

Looking ahead, JKBNS must now pursue indexing in PubMed Central and the Emerging Sources Citation Index. Key steps include increasing the number of English-language publications [2], diversifying the nationalities of authors and editorial board members, reducing reliance on submissions from board members, and actively recruiting external contributors. These measures will enhance credibility, accessibility, and search functionality while ensuring adherence to publication ethics [3].

To further improve its quality, JKBNS must meet various evaluation criteria, including the academic value of the journal according to the Web of Science Group, citation metrics for authors and editorial board members, and comparison with other journals. Authors and editorial board members must fulfill the citation network and category relevance requirements in Web of Science. The content of the journal must possess originality, applicability, and both regional and international significance. Strategic selection of high-impact papers and collaborative efforts to boost the journal’s impact factor will be essential [4].

Finally, KSBNS members must channel their expertise into efforts to advance both education and research in order to improve the organization. In terms of education, the content of biological nursing science must be closely linked to clinical practice, and efficient teaching methods must be developed to maximize educational effectiveness. Researchers must prioritize studies employing advanced biological measurements and laboratory experiments, thereby expanding nursing’s scientific foundation. These efforts in education and research will drive the advancement of biological nursing science, contribute to the development of the foundational disciplines of nursing, and ultimately support the scientific development of the field.

The indexing of JKBNS in Scopus represents a transformative moment for the KSBNS. By uniting to elevate the journal’s international profile and rigor, members can ensure its continued growth. I sincerely congratulate all involved on this historic achievement and look forward to the society’s ongoing contributions to nursing science worldwide.

## Data Availability

None.

## References

[1] Lee KE, Park YR, Cho KJ, Park MJ (2011). Research trends in the Korean biological nursing science -based on analysis of the research papers published in the Journal of Korean Biological Nursing Science from 1999 to 2010-. Journal of Korean Biological Nursing Science.

[2] Huh S Inclusion of PubMed Central journals in MEDLINE and the status of Korean and bilingual MEDLINE journals in PubMed Central [Internet]. https://www.kamje.or.kr/webzine/view_webzine?webzine_id=2021-6-76&se_id=1.

[3] Korean Association of Medical Journal Editors Comprehensive evaluation and reflections from the 51st new evaluation meeting [Internet]. https://www.kamje.or.kr/webzine/view_webzine?webzine_id=2024-12-90&se_id=1.

[4] Huh S Challenges and opportunities in achieving inclusion in the Emerging Sources Citation Index (ESCI) [Internet]. https://www.kamje.or.kr/webzine/view_webzine?webzine_id=2020-6-72&se_id=5.

